# E-health Dietary Interventions for Participants of SNAP and WIC: A Systematic Review

**DOI:** 10.1016/j.cdnut.2024.102099

**Published:** 2024-02-08

**Authors:** Mayra Crespo-Bellido, Josephine Fernandez Ong, Amy Yaroch, Carmen Byker Shanks

**Affiliations:** 1 Gretchen Swanson Center for Nutrition, Omaha, NE, United States; 2 School of Health Sciences, Northern Illinois University, DeKalb, IL, United States

**Keywords:** e-health, m-health, dietary intervention, SNAP, WIC

## Abstract

The migration of federal assistance services to online platforms during the COVID-19 pandemic sparked interest in digital nutrition education for individuals participating in the Supplemental Nutrition Assistance Program (SNAP) and Special Supplemental Nutrition Program for Women, Infants, and Children (WIC) programs. With federal government investing in the modernization of the nutrition education components of both programs, there is a need to identify science-backed electronic health (e-health) dietary interventions to improve health outcomes in this population. Therefore, the objective of this systematic literature review was to summarize the effectiveness, acceptability, and feasibility of e-health dietary interventions among individuals participating in WIC or SNAP. Keyword searches were performed in Google Scholar, PubMed, and Science Direct. The search included peer-reviewed literature from 2014 to 2023 and a few articles offering context about interventions used long-term by the nutrition assistance programs. PRISMA guidelines were followed to conduct this systematic literature review, which resulted in 36 articles eligible for extraction. The studies evaluated e-health (52.8%), short message service/text messaging (27.8%), and smartphone application interventions (19.4%) delivered to WIC or SNAP participants. The interventions identified aimed to modify food choice, eating behavior, and dietary intake among SNAP participants, SNAP-eligible adults, and WIC participants. Most interventions were developed using content delivery and health behavior theoretical frameworks (77.8%) and evidence-based nutritional recommendations (59.3%). Review findings show a high level of acceptability and feasibility for e-health and mobile health dietary interventions among WIC and SNAP participants but varying levels of effectiveness. Level of engagement, dosage, retention, and adherence were strong predictors of positive dietary behavior change regardless of the mode of intervention delivery. Future studies need to prioritize health equity by recruiting samples representative of food nutrition assistance participants and addressing digital health literacy as a potential barrier to intervention effectiveness, as none of the present studies measured literacy among participants.

## Introduction

The USDA Food and Nutrition Service (FNS) administers federal food assistance programs, which are designed to support food and nutrition security for Americans with lower income. In 2022, the Supplemental Nutrition Assistance Program (SNAP), which is the largest federal food assistance program in the United States, served 41.2 million participants, and the Special Supplemental Nutrition Program for Women, Infants, and Children (WIC) served 6.2 million participants [1,2].

In addition to providing support to purchase food, these 2 wide-reaching federal food assistance programs provide resources to develop and deliver high-quality, evidence-based nutrition education to individuals with low income through various mechanisms. Within the SNAP program, state agencies have the option to deliver nutrition education through SNAP Education (SNAP-Ed) via contracts with SNAP-Ed implementing agencies [3]. State agencies select food and nutrition programming that aligns with the latest Dietary Guidelines for Americans (DGA), aiming to enhance the dietary habits of SNAP participants and individuals with incomes lower than 185% of the federal poverty line [3]. However, only a subset of SNAP participants actively receives SNAP-Ed programming. Alternatively, food and nutrition education to improve dietary behavior is woven into WIC programming. Through WIC Works, FNS provides resources for WIC state and local clinic staff to provide relevant resources to participants [4]. However, WIC state agencies can choose and create nutrition education content that aligns with federal WIC nutrition education guidelines and policies.

The COVID-19 pandemic altered all aspects of daily life, including federal government services, which adapted to serve participants in the face of social distancing regulations and spikes in food insecurity [5]. Traditionally, SNAP-Ed and WIC offered nutrition education face-to-face in clinical and community settings. However, since the onset of the pandemic, many healthcare services have been offered online, requiring individuals participating in federal nutrition assistance to adapt to new technologies to receive health assessments and nutrition education services. As federal programs’ pandemic protections unwind, some participants have expressed preferences for hybrid methods that allow some services to remain online [6,7], including food and nutrition education.

The promise of digital interventions to deliver nutrition care to individuals with lower income has been evaluated in the literature [8]. Studies found similar interest from individuals participating in SNAP and WIC for receiving nutrition education, preferably via e-mail, Facebook, and text messaging compared with in person [[9], [10], [11]]. Issues with health literacy, transportation issues, and costs of missing work have limited the delivery of in-person nutrition education for individuals participating in SNAP and WIC [[9], [10], [11]]. Moreover, the increased accessibility to the Internet and everyday information-seeking via websites, social media, and smartphone applications (apps) have made it feasible for healthcare professionals to reach participants of federal food assistance programs online. According to the Pew Research Center, in 2021, among individuals with an annual income lower than $30,000, 97% own a cellphone, 76% own a smartphone, and 93% have access to the Internet [12,13]. Social media is also a known important source of health information and misinformation for United States adults. The percentage of United States individuals who have reported using social media has increased from 5% in 2011 to 72% in 2021, with Facebook being the most popular among individuals with yearly income lower than $30,000 (70%) [14].

The federal government has reaffirmed their commitment to the ongoing modernization of SNAP and WIC, with a particular emphasis on centering equity in these efforts. In January of 2023, USDA announced an investment of $25 million for pilot projects that offer electronic health (e-health) incentives to individuals participating in SNAP [15]. As part of the American Rescue Plan Act, FNS received $390 million for outreach, innovation, and modernization of WIC. As state agencies continue to offer services to online or hybrid methods, there is a need for evidence-based dietary interventions that are appropriate for populations with differing health status, diverse cultural backgrounds, and varying digital literacy skills. These digitally based dietary interventions should also consider social determinants of health that may impede meaningful health behavior change in populations with low socioeconomic status. The objective of this systematic literature review is to summarize the effectiveness, acceptability, and feasibility of e-health dietary interventions in improving food and nutrition outcomes among individuals participating in WIC or SNAP.

## Methods

This systematic review follows the recommended protocols from the Johanna Briggs Institute (JBI) and PRISMA. Based on JBI guidelines [16], the following research question was developed using the “Population, Concept, and Context” framework: What is the effectiveness, acceptability, and feasibility of e-health or mobile health (m-health) interventions in modifying dietary behaviors (concept) among federal food assistance participants (population) in SNAP and WIC (context)? In answering this research question, the goal is to identify best practices and future directions for e-health interventions for SNAP and WIC participants. Preliminary searches using the Cochrane Database of Systematic Reviews, PROSPERO, the JBI Evidence Synthesis, and Google Scholar were conducted, and no current or underway systematic reviews or scoping reviews on the present topic were identified. The JBI critical appraisal tools for the respective methodology of the study were used to assess the methodological quality of the included articles [[17], [18], [19], [20]]. JBI critical appraisal tools have a different number of items, from 8 items for cross-sectional studies to 13 items for randomized controlled trials (RCTs), and possible answers are “yes,” “no,” “unclear,” and “not applicable” [[17], [18], [19], [20]]. Because the tools have variable number of items, to compare scores, we calculated what percentage of answers were “yes” out of the total number of items and graded them as good quality (>80%), fair quality (60%–80%), and low quality (<60%). Mixed-methods studies were appraised using the cross-sectional tool and the qualitative study tool and calculating an average percentage.

### Search strategy

In 2022, 1 author (MCB) identified search terms based on keywords used in existing publications on e-health dietary interventions. These initial keywords including “e-health/electronic health interventions among SNAP participants,” “online nutrition interventions among SNAP or WIC participants,” and “m-health/mobile health interventions among SNAP and WIC participants” were used to identify relevant literature in PubMed and Google Scholar. Only peer-reviewed articles were included in the initial search. Three databases, PubMed, ScienceDirect, and Google Scholar, were pretested and selected to identify peer-reviewed sources relevant to the review scope. A second search was conducted in June 2023 to identify new articles which emerged since then (only including the year 2023). The search strategy approach and key terms for each database are shown in Table 1. In addition, the references of all articles included in full-text extraction were reviewed to identify additional sources meeting inclusion criteria that may have been missed through database searches. Finally, results of current systematic or scoping reviews on e-health/m-health interventions among individuals with a low income were compared with the search strategy results. Any sources not captured by the initial database search were scanned for relevance for inclusion in this review.

**TABLE 1:** Peer-reviewed literature search strategy

PubMed	(("ehealth"[Title/Abstract] OR "e-health"[Title/Abstract] OR "mhealth"[Title/Abstract] OR "m-health"[Title/Abstract] OR "e-learning"[Title/Abstract] OR "online"[Title/Abstract] OR "mobile health"[Title/Abstract])) AND ("intervention"[Title/Abstract] OR "RCT"[Title/Abstract] OR "clinical trial"[Title/Abstract])) AND (SNAP[Title/Abstract] OR WIC[Title/Abstract])
ScienceDirect and Google Scholar	("e-health" OR "ehealth" OR "m-health" OR "mhealth" OR "online" OR "web-based" OR "elearning" OR “smartphone app”) AND ("SNAP" OR "WIC") AND ("intervention")

### Eligibility criteria

Articles testing the effectiveness, acceptability, and feasibility of an e-health or m-health intervention to change dietary behaviors among SNAP, SNAP-eligible, or WIC participants were included. All types of research designs published in peer-reviewed journals within the past 10 y were included. Two articles published >10 y ago were included because they offered context about an intervention that was published in the last 10 y, wichealth.org, which has been used by WIC for an extended period. Studies were excluded if they were non–peer-reviewed, the outcome measure was not dietary behavior-related, or the intervention tested was a social marketing campaign.

### Interventions of interest

For this systematic literature review, e-health is defined as health services and information delivered or enhanced through the Internet and related technologies [21]. M-health is a category of e-health that is delivered via smartphone apps, text, or short message service (SMS) messaging. More specifically, e-health dietary interventions are defined as purposefully planned actions, counseling and resources developed to improve dietary behaviors (i.e., food choice, eating behaviors, and dietary intake) partially or completely delivered online or via text messaging [22]. We included interventions that had in-person components paired with e-health, such as support groups or produce bags. It should be noted that food choice, eating behaviors, and dietary intake represent different aspects of dietary behavior [22]. Food choice refers to behaviors before consumption, including choosing based on food preferences, the share of income spent on food, willingness to pay, frequency of purchase, product purchase, food preparation, and intentions to choose a specific food [22]. Eating behaviors occur during consumption and include eating habits, parental feeding practices, eating occasions, food portions, dieting, disordered eating symptoms (under- or overeating), and neophobia, pickiness, or fussiness [22]. Dietary intake is the outcome of food consumption, including anything related to changing dietary pattern, meal pattern (e.g., meal content, energy and nutrient distribution), and food intake (including breastfeeding) [22].

### Implementation outcomes of interest

We included studies that addressed effectiveness, acceptability, and feasibility of the intervention. Effectiveness is the extent to which an intervention or treatment achieves its intended goals and produces the desired outcomes [23]. It assesses the actual impact and success of the intervention. The current study defines acceptability as the “perception among implementation stakeholders (e.g., implementers, users) that a given treatment, service, practice, or innovation is agreeable, palatable, or satisfactory” [23,24]. Feasibility is the “extent to which a new treatment, or innovation, can be successfully used or carried out within a given agency or setting” [23]. It considers factors, such as resource availability, logistical constraints, and the ability to carry out the intervention in real-world settings.

### Data extraction

Citations were exported to Covidence (Veritas Health Innovation) for screening and extraction. Duplicates were automatically removed, and titles and abstracts were screened for eligibility by 2 reviewers (MCB and JFO). Two reviewers (MCB and JFO) reviewed full-text articles for eligibility, and 1 reviewed the articles’ quality and extracted the relevant information into an extraction template (MCB). Any disagreement was resolved by a third reviewer (CBS).

Data were extracted from the eligible articles to a Microsoft Excel spreadsheet adapted from a previous systematic review. The data collected included author, year, study objective, research design, state, community setting (urban, rural, or combination), SNAP/SNAP-eligible or WIC population, sociodemographic characteristics of the sample, sample size, name of the intervention, type of intervention (m-health, e-health), mode of delivery, the theoretical framework for intervention development, nutrition evidence or guidelines for intervention development, outcomes measured (e.g., anthropometric outcomes, food intake, knowledge, attitudes, and behaviors impacted by dietary intervention), and relevant findings. The charted data was summarized as counts when possible.

## Results

**FIGURE 1:**
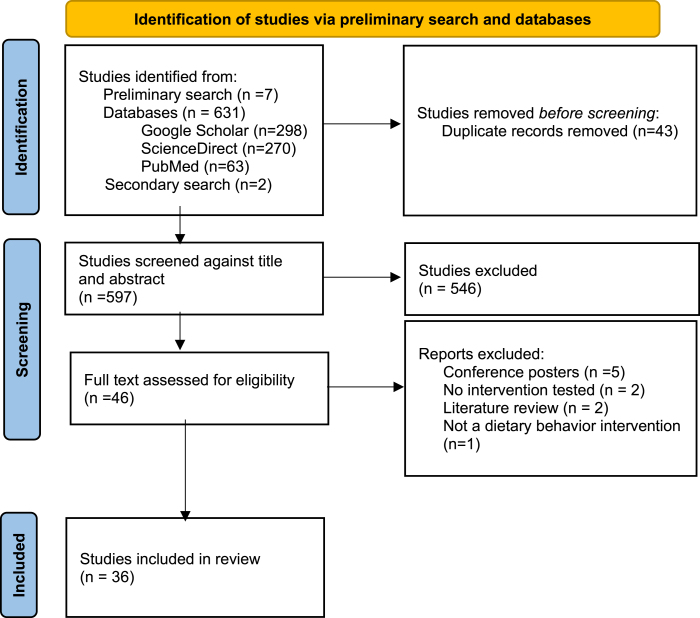
Screening process using PRISMA flowchart.

Seven studies were identified in the preliminary search stage; 631 were identified from the databases PubMed, Google Scholar and ScienceDirect, and 2 were identified in a secondary search prior to publication. The software, Covidence, removed 43 duplicates, and 546 articles were excluded based on the title and abstract screening. Among the 46 full-text articles assessed for eligibility, 5 were not peer-reviewed, 2 were not e-health/m-health interventions, 2 were not on the population of interest, and 1 was not a dietary behavior intervention. Figure 1 presents the screening process using PRISMA flowchart.

### Study characteristics

All studies (*n* = 36) were published between 2014 and 2023, except for 2 supporting wichealth.org articles, which were older. Approximately 64% (*n* = 23) of the studies examined interventions among WIC participants or caregivers, 31% (*n* = 11) were evaluations among SNAP participants or SNAP-eligible individuals, and 5% (*n* = 2) tested interventions with both SNAP and WIC participants (Table 2). Sample sizes ranged from 9 participants in a qualitative app beta-testing study to 305,735 participants in a cross-sectional study.

**TABLE 2:** Characteristics of studies on dietary interventions for SNAP and WIC participants

Characteristics	Overall (*n* = 36)		SNAP (*n* = 11)		WIC (*n* = 23)		SNAP and WIC (*n* = 2)	
	*n*	%	*n*	%	*n*	%	*n*	%
Sample sizes, median (range)	170.5 (9–305,735)	—	115 (20–461)	—	174 (9–305,735)	—	272.5 (257–288)	—
Study design								
RCT	12	33.3%	1	9.1%	10	43.5%	1	50.0%
Cluster RCT	4	11.1%	1	9.1%	3	13.0%	0	0.0%
Cross-sectional study	5	13.9%	1	9.1%	4	17.4%	0	0.0%
Quasiexperimental design	7	19.4%	3	27.3%	3	13.0%	1	50.0%
Qualitative	4	11.1%	2	18.2%	2	8.7%	**0**	0.0%
Mixed-methods study	4	11.1%	3	27.3%	1	4.3%	0	0.0%
Type of intervention								
E-health	19	52.8%	7	63.6%	10	43.5%	2	100.0%
M-health								
App	7	19.4%	2	18.2%	5	21.7%	0	0.0%
Text/SMS	10	27.8%	2	18.2%	8	34.8%	0	0.0%
Dietary behavior and target population								
Dietary intake (*n* = 10)								
Children (C)	6	16.7%	1	9.1%	5	21.7%	0	0.0%
Parents (P)	2	5.6%	0	0.0%	2	8.7%	0	0.0%
Adults (A)	2	5.6%	2	18.2%	0	0.0%	0	0.0%
Eating behavior (*n* = 6)								
Children (C)	3	8.3%	0	0.0%	3	13.0%	0	0.0%
Parents (P)	2	5.6%	0	0.0%	2	8.7%	0	0.0%
Adults (A)	1	2.8%	1	9.1%	0	0.0%	0	0.0%
Food choice (*n* = 7)								
Household (HH)	7	19.4%	3	25.0%	4	17.4%	0	0.0%
Multiple behaviors (*n* = 11)								
Eating behavior (HH), Dietary intake (HH)	1	2.8%	0	0.0%	1	4.3%	0	0.0%
Food choice (HH), Dietary intake (C)	1	2.8%	0	0.0%	1	4.3%	0	0.0%
Food choice (HH), Dietary intake (HH)	1	2.8%	1	8.3%	0	0.0%	0	0.0%
Food choice (A), Dietary intake (A)	3	8.3%	3	25.0%	0	0.0%	0	0.0%
Food choice (P), Dietary intake (P)	5	13.9%	0	0.0%	5	21.7%	1	50.0%
Food choice (P), Eating behavior (P)	1	2.8%	0	0.0%	0	0.0%	0	0.0%
Food choice (P), Dietary intake (C)	1	2.8%	0	0.0%	0	0.0%	1	50.0%

Abbreviations: App, smartphone application; RCT, randomized control trial; SMS, short message service; SNAP, Supplemental Nutrition Assistance Program; WIC, Special Supplemental Nutrition Assistance Program for Infants Women, and Children.

Twelve of the studies were RCTs, followed by 7 studies with quasiexperimental design (e.g., convenience placement in study condition, or comparing the pre- and postintervention surveys). Cluster RCT studies (*n* = 4) randomized WIC clinics or sites to the intervention or standard care groups (e.g., standard WIC nutrition education or 1 of the SNAP-Ed curricula). Cross-sectional interventions gleaned administrative and/or survey data from e-health dietary interventions implemented system wide. This method was possible for the wichealth.org intervention, which has been used in WIC for 20 y [25], and the WICShopper app which is used in 32 states, 3 American-Indian tribes, and 1 United States territory [26].

The studies included had an overall mean quality score of 81%. Out of these, 21 were categorized as good quality [[27], [28], [29], [30], [31], [32], [33], [34], [35], [36], [37], [38], [39], [40], [41], [42], [43], [44], [45], [46], [47]], 13 as fair quality [[48], [49], [50], [51], [52], [53], [54], [55], [56], [57], [58], [59], [60]], and 2 as low quality [61,62]. Among the 2 fair-quality cross-sectional studies, there were issues with identifying and addressing confounding factors, and the validity and reliability of outcome measurements were unclear [55,56]. Similarly, the 8 fair-quality RCTs often lacked clarity in terms of concealing treatment group allocation, participant blinding, and proper analysis of attrition differences [[48], [49], [50], [51], [52], [53], [54],^59^]. The 3 quasiexperimental studies were rated as fair quality due to the absence of a control group and a lack of attrition analysis [57,58,60]. Finally, the 2 low-quality RCTs failed to specify their randomization method, lacked information on participant and assessor blinding, and did not describe attrition differences between the treatment and control groups [61,62].

E-health interventions (with no mobile phone use) were evaluated in slightly over half of the studies, whereas m-health interventions were further split into text/SMS interventions that required access to text messaging services (*n* = 10) and app interventions that required access to a compatible smartphone or tablet device (*n* = 7). Interventions were all conducted among adults. However, some studies examined nutritional outcomes among varying groups: 9 were among children, 7 among parents or adults, and 20 among >1 household member.

Several of the studies were implemented in racially and ethnically diverse populations tied to many different cultures. Yet, few discussed culturally tailoring materials (e.g., language, foods) or the implications of a diverse sample. One study compared the Fit Moms/Mamás Activas app engagement between Spanish- and English-speaking Hispanic females. Females who used the English version engaged more with the content than the Spanish version [49]. In the development of the WIC Fresh Start intervention, Spanish-speaking WIC participants were consulted for feedback on the modules and recruited to narrate the audio for the videos, which led to greater voucher redemption among participants [63]. For the SMS intervention, developed in collaboration between the University of Hawaii (UH) and University of Puerto Rico (UPR), materials were culturally tailored in English for the Hawaiian participants and Spanish for the Puerto Rican participants [52,53]. Two interventions were administered to predominantly Hispanic samples in English and Spanish, but there was no discussion about how the educational materials were tailored [61,62]. The Food eTalk intervention was tailored to use a Southern accent and present healthy preparations of Southern dishes [[42], [43], [44]].

### Characteristics of e-health interventions

Table 3 [[28], [29], [30], [31], [32], [33], [34], [35], [36], [37],^40^,^41^,^45^,[47], [48], [49], [50], [51], [52], [53],[60], [61], [62]], Table 4 [27,38,[42], [43], [44],^46^,[55], [56], [57], [58], [59]], and Table 5 [39,54] present the 27 unique interventions evaluated: 10 among SNAP participants or SNAP-eligible individuals, 15 among WIC participants, and 2 among SNAP and WIC participants. Most interventions (78%) were created with a content delivery theoretical framework (e.g., Knowles et al. Adult Learning Theory) and/or with a health behavior theory (e.g., Health Belief Model) [[28], [29], [30],[33], [34], [35], [36], [37], [38], [39], [40], [41], [42], [43], [44], [45],[47], [48], [49],[52], [53], [54], [55], [56],[58], [59], [60], [61], [62]]. Although most interventions (70%) among SNAP-eligible individuals or participants reported using nutritional guidelines (e.g., DGA, Dietary Approaches to Stop Hypertension) to develop their content [38,[42], [43], [44],^46^,^56^,^57^,^59^], over half of the interventions for WIC participants did not specify using nutritional guidelines to design their interventions [28,[31], [32], [33],^40^,^41^,[47], [48], [49], [50],^52^,^53^]. Interventions intended for SNAP participants mostly aimed to modify adults’ dietary practices: 55% of SNAP (*n* = 6) interventions aimed to only modify the behaviors of the adult participating in the study, 36% of SNAP interventions (*n* = 4) aimed to modify the dietary behaviors of >1 household member, and only one SNAP intervention was intended for children. Notably, 35% of WIC interventions (*n* = 8) were intended to modify the dietary behavior of children, 39% intended to modify the dietary behavior of parents (*n* = 9), and 26% were intended for >1 household member.

**TABLE 3:** Summary of studies on e-health and m-health dietary interventions among WIC participants

Intervention name (type)	Citation	Study objective	Sample characteristics	Dosage^1^	Intervention setting and delivery	Relevant findings
Breakfast Class (e-health)	Au [61], 2016	Test the effectiveness of online intervention vs. traditional education on improving breakfast intake.	Women with low-income (*n* = 591)	8–16 wk from pre- to posttest; 1 online class	WIC staff taught class in-person at the WIC clinic and online module mirrored class content and visuals. Participants recruited at WIC clinics.	Online group parents and their children had increases in breakfast frequency compared with the in-person group.
Children Eating Well (CHEW) [m-health (app)]	Hull [47], 2017	Understand the feasibility and acceptability of an app to improve child feeding practices.	Black and Hispanic mothers of children enrolled in WIC (*n* = 63)	13 wk; once a week for 4.5 min on average	Research team made in-home visits to install mobile app in participants’ phone. Participants recruited from a list of WIC participants.	Participants used the app on average 1/wk for 4 min; using the shopping related features 1–2/mo and the snack gallery 2–4/mo. Feedback indicated the need for improving WIC Shopping Tools functionality.
Cooking Matters Facebook page (e-health)	Zhang [40], 2021	Test the effectiveness of exposure to a dietary intervention’s social media page on improving healthy meal preparation.	WIC caregivers and WIC-eligible caregivers (*n* = 167)	8 wk; Facebook algorithm determined exposure to content	Research team delivered intervention via Facebook page. Participants were recruited via a banner in the WICShopper app.	No change in most measurements at follow-up except in self-efficacy to provide a healthy meal within a budget. Some subgroups improved attitudes toward cooking (e.g., men and single-headed households) and healthy meal preparation (e.g., younger caregivers).
Fit Moms/Mamás Activas (e-health)	Bennion [33], 2020	Test the effectiveness of an online intervention compared with WIC standard care in producing weight control behaviors.	postpartum women with low income (*n* = 370)	52 wk; 52 weekly web-based lessons, 365 weight monitoring prompts and 208 text messages	Research team delivered the intervention (including monthly group meetings at WIC clinic). Participants recruited at WIC clinics.	The online intervention resulted in greater increases in weight control behaviors, self-monitoring of weight and eating, and cognitive restraint compared with standard care.
	Phelan [48], 2017	Test the effectiveness of an online intervention compared with WIC standard care in producing weight loss.	postpartum females with low income (*n* = 370)	52 wk; 52 weekly web-based lessons, 365 weight monitoring prompts and 208 text messages		The online intervention group produced greater mean 12-mo weight loss than the standard care group. More participants in the intervention group than in the standard care group returned to preconception weight by 12 mo.
	Power [49], 2019	Test the effectiveness of an online intervention in weight loss by the level of engagement with content.	postpartum females with low income in the intervention arm of the study (*n* = 174)	52 wk; 52 weekly web-based lessons, 365 weight monitoring prompts, and 208 text messages		More frequent engagement with the web diary and discussion forum and greater attendance at in-person group sessions predicted greater percent weight loss at 12 mo. Women who used the English version engaged more with the content than the predominantly Spanish-speaking females.
Healthy4Baby [m-health (text)]	Herring [50], 2014	Understand the feasibility, acceptability, and initial efficacy of a weight loss intervention with a texting and social media component.	racially diverse, postpartum females with low income (*n* = 18)	14 wk; 98 messages, 28 calls with a health coach, and 3 monthly raffles announced on Facebook	Research team delivered Intervention through Facebook. Usual care delivered through WIC staff or primary care providers. Participants recruited at outpatient clinics (obstetrics, pediatrics).	The technology-based intervention participants had significantly greater weight loss than usual care. One-third of intervention participants and no control participants lost >5% of their initial body weight at follow-up.
Healthy Roots [m-health (text)]	Kay [60], 2023	Understand the feasibility and acceptability of a text messaging intervention to support redemption and consumption of WIC-approved foods to ultimately improve diet quality.	Dyads of WIC adult caregivers with children aged 0–2 y (*n* = 54)	12 wk; 36–48 text messages	Research team delivered intervention via text messaging. Participants recruited at WIC clinics.	Half of the caregivers replied to 80% or more of the text messages, and over a quarter of participants replied to all the text prompts. Most felt the nutrition feedback and tips were helpful and would recommend the program. More were consuming leafy green vegetables compared with baseline.
Lactation Advice Through Texting Can Help (LATCH) [m-health (text)]	Martinez-Brockman [35], 2018a	Test the effectiveness of a 2-way text messaging intervention to improve breastfeeding outcomes.	Women with low income participating in breastfeeding peer counselor program (*n* = 174)	13 wk; 2-way texting (a minimum of ∼170 1-way texts)	Breastfeeding peer counselors delivered the intervention via text messaging and usual care in person for the control group. Participants recruited at WIC clinics.	The text intervention had a significant impact on early contact between participants and breastfeeding peer counselors but did not have a significant impact on exclusive breastfeeding.
	Martinez-Brockman [36], 2018b	Test the effectiveness of a 2-way text messaging intervention to improve breastfeeding outcomes by level of engagement.	Women with low income participating in breastfeeding peer counselor program (*n* = 70)	13 wk; 2-way texting (a minimum of ∼170 1-way texts)		The intensity of engagement during the first 2 wk postpartum was the strongest predictor of exclusive breastfeeding status.
	Martinez-Brockman [45], 2020	Content analysis of text discussions among high engagement participants in a 2-way text messaging intervention.	Women with low income participating in breastfeeding peer counselor program (*n* = 54)	13 wk; 2-way texting (a minimum of ∼170 1-way texts)		High engagement participants discussed the mechanics of breastfeeding, social support, and infant nutrition. In the postpartum period they discussed breastfeeding problems and complications.
MomLink [m-health (app)]	Chaudhry [41], 2019	Understand the feasibility and usability of an app to improve healthy eating and adequate pregnancy care.	Women with low income in reproductive age (*n* = 9)	1 d; 1 time exploration of the app	Research team delivered the intervention at WIC clinics. Participants recruited at WIC clinics.	Most tasks were completed without help, and high usability scores, but low engagement. Usability problems: unclear icons, unmet expectations, unclear goals, confusing layout.
Salt Education Class (e-health)	Au [62], 2017	Test the effectiveness of online intervention vs. traditional education on salt intake.	Women with low-income (*n* = 514)	39–48 wk from pre- to posttest; 1 class	WIC staff taught class in-person at the WIC clinic and online. module mirrored class content and visuals. Participants recruited at WIC clinics.	Positive changes in knowledge, self-efficacy, and sodium reduction were retained at follow-up later for both groups; no difference between groups.
SmartLoss app [m-health (app)]	Gilmore [51], 2017	Test the effectiveness of app intervention to promote weight loss and improve diet quality.	postpartum WIC participants with overweight and obesity (*n* = 40)	16 wk; daily weight monitoring and 1 weekly push notification	Research team delivered intervention in app and WIC clinic, WIC staff delivered usual care for control group. Participants recruited at WIC clinics.	Only participants with high intervention adherence had a significant reduction in body weight and percent body fat when compared with standard care; overall, there was no difference between groups.
SMS developed by UH [m-health (text)]	Holmes [34], 2020	Test the effectiveness of texting intervention on excessive gestational weight gain.	WIC participants with overweight and obesity at 15–20 wk pregnant (*n* = 83)	18 wk; 18 weekly text messages	Research team delivered the intervention via third-party web-based text messaging platform. Participants recruited at WIC clinics.	Gestational weight gain was not significantly different between intervention and control groups.
SMS developed by UPR and UH [m-health (text)]	Gibby [52], 2019	Measure the acceptability of a text message-based intervention for prevention of excessive infant weight gain.	Mothers with infants ages 0–2 mo at baseline (*n* = 80)	18 wk; 18 weekly text messages	Research team delivered the intervention via third-party web-based text messaging platform. Participants recruited at WIC clinics.	Most participants reported that they liked all the messages, that they were all useful and that they led them to make changes in the way they fed their infants.
	Palacios [53], 2018	Test the effectiveness of a SMS intervention to improve infant feeding practices and infant weight.	WIC caregivers of healthy 0–2-mo infants (*n* = 202)	18 wk; 18 weekly text messages		There was no difference in weight status, weight gain rate, nor child feeding practices between groups.
WIC Fresh Start (e-health)	Di Noia [28], 2017	Test the effectiveness of an online intervention compared with traditional education on FV intake and FV voucher redemption.	WIC enrolled females (*n* = 744)	13 wk; 3 modules	Research team delivered the intervention at a WIC clinic and online. Participants recruited at a WIC clinic.	FV intake did not differ between groups. Online intervention was associated with FMNP voucher redemption (in Spanish-speaking participants); improvements in knowledge, purchasing behaviors, and FV intention at a farmers’ market; FV food safety and preparation skills.
wichealth.org (e-health)	Bensley [29], 2006	Test the effectiveness of an online nutrition education website to improve child feeding practices.	WIC caregivers (*n* = 39,541)	no timeframe; ≥1 of 5 online modules	Online WIC program education platform. WIC administrative data used.	Movement in stage was greatest for the module on “picky eating.” Contemplation as the beginning stage had the greatest stage movement. Self-efficacy to engage in behavior was associated with 7 of the 8 modules (all except breastfeeding).
	Bensley [37], 2011	Test the effectiveness of online intervention vs. traditional education on FV intake.	WIC caregivers (*n* = 691) and children (*n* = 872)	39 wk; 2 modules		The Internet group experienced positive differences in the stage of change progression, the perception that the intervention was helpful and easy to use, and FV consumption.
	Brusk [30], 2016	Comparing the impact mobile vs. fixed devices have on user engagement with an online intervention.	WIC participants using wichealth.org (*n* = 305,735)	no timeframe; ≥1 of 5 online modules		Nonmobile users were more likely to engage based on all 3 key performance indicators.
WICShopper [m-health (app)]	Zhang [31], 2020	Test the effectiveness of an app to improve the redemption of the prescribed food packages.	WIC participants and caregivers (*n* = 30,440)	4 wk; user chose engagement	Mobile app used by WIC program. WIC administrative data used.	App users consistently had higher average redemption rates than nonapp users. More active cycles and active days in the cycle were significantly related to redemption rates for all categories, except for frozen juice.
	Zhang [32], 2021	Examine the relationship between app usage and full redemption of the prescribed food packages.	WIC participants and caregivers (*n* = 23,050)	4 wk; user chose engagement		App users had a higher prevalence of full redemption in most food categories (except infant meats, infant formula, and legumes).

Abbreviations: App, smartphone application; FMNP, Farmers Market Nutrition Program; FV, fruits and vegetables; SMS, short message service; UH, University of Hawaii; UPR, University of Puerto Rico; WIC, Special Supplemental Nutrition Assistance Program for Infants Women, and Children.

1 Dosage includes study length and contact occasions.

**TABLE 4:** Summary of studies on e-health and m-health dietary interventions among SNAP participants

Intervention name (type)	Citation	Study objective	Sample characteristics	Dosage	Intervention setting and delivery	Relevant findings
Adapted Food for Thought: Eating Well on a Budget (e-health)	Lawton [46], 2022	Understand the feasibility of a social media intervention to improve food resource management and healthy eating on a budget.	SNAP-eligible parents of children in Head Start (*n* = 30)	3 wk; 31 Facebook posts	Researchers delivered the intervention via Facebook. Participants were recruited from Head Start.	Facebook was found to be a feasible platform for delivering the intervention; parents were engaged throughout the intervention. Discussion questions had the highest level of interaction.
Cooking Matters App [m-health (app)]	Garvin [55], 2019	Measure the acceptability and effectiveness of an app to improve meal planning and preparation features.	Parents and caregivers with low income (*n* = 461)	no timeframe; user chose engagement	Nonprofit organization delivered intervention via mobile CM app. Participants recruited via the app, at WIC clinics, and a Head Start national conference.	Mean responses were positive toward “meal planning” and “shopping and cooking.” Interviewees described meal planning and preparation behaviors as intrinsic, based on habit, and influenced by family preference and food costs.
Food eTalk (e-health)	Stotz [42], 2018	Understand the feasibility and acceptability of an online Nutrition Education Program.	SNAP-Ed−eligible Georgian adults (*n* = 61)	13 wk; 11 eLearning nutrition education lessons	Research team delivered intervention via a website. Participants recruited from Head Start programs, public libraries, parenting support groups, General Education Diploma programs, safety-net clinics and faith-based organizations.	Acceptability of the intervention was high. Participants discussed potential barriers, such as motivation to engage, and solutions, like improved program content (e.g., gamification of content and interactive short videos).
Food eTalk: Better U (e-health)	Stotz [44], 2019	Test the effectiveness of an online intervention and produce prescriptions to promote healthy eating.	SNAP-Ed−eligible Georgian adults (*n* = 26)	13 wk; 11 eLearning lessons (plus FV provided)	Safety-net clinic staff delivered the produce bags at the clinic and participants accessed the lessons online in their loaned smartphones. Participants were recruited at the safety-net clinic.	Quantitative data analysis did not show significant differences between the intervention and comparison groups in pre- and postclinical and anthropometric measures.
	Stotz [43], 2021	Measure the acceptability: To describe a participant-driven, text message-based social support network that emerged organically from an online intervention.	SNAP-Ed−eligible Georgian adults (*n* = 20)	13 wk; 11 eLearning nutrition education lessons		Participants initiated a robust group text message support system through which they shared encouragement, recipes, grocery shopping tips, and images of food they prepared with the produce box.
Fresh Conversations (e-health)	Wong [56], 2022	Measure the acceptability of an online intervention to promote nutrition, food safety, food security, and physical activity.	SNAP-eligible older adults (*n* = 115)	16 wk; 4 meetings and 4 newsletters	Extension and public health department staff delivered the intervention via videoconference and teleconference platforms. Participants were recruited remotely from existing in-person intervention participants, Area Agencies on Aging, and from senior affordable housing.	Overall, participants were “satisfied/very satisfied,” learned something new, and intended to make behavior change; the group detected no differences in satisfaction or reported impacts.
Lessons developed by the UN’s SNAP-Ed program (e-health)	Campbell [57], 2013	Test the effectiveness of a distance nutrition education intervention compared with the traditional face-to-face delivery method.	SNAP-eligible participants (*n* = 213)	no timeframe; ≥2 of 6 lessons	SNAP-Ed and Expanded Food and Nutrition Education Program (EFNEP) staff delivered the program in person and remotely (online, mail). Participants were recruited from SNAP-Ed and EFNEP sites.	Both groups showed increased positive behavior (food resource management, nutrition practices, food safety practices) and nutrient intake changes of a similar magnitude.
MyQuest [m-health (text)]	Griffin [38], 2018	Test the effectiveness of a texting intervention to improve dietary behaviors and weight management.	SNAP-eligible, overweight or obese females with low income (*n* = 104)	12 wk; 168–252 texts and 12 electronic newsletters	Research team delivered intervention via text messaging. Participants recruited from SNAP office, the Department of Housing and Urban Development, and a food bank.	Postintervention participants significantly improved dietary and physical activity behaviors, increased dietary and physical activity goal setting, and reduced weight.
Text2BHealthy [m-health (text)]	Grutzmacher [58], 2018	Test the effectiveness of an SMS-based intervention to improve school children’s FV intake.	SNAP-eligible caregivers with low-income (*n* = 142)	52 wk; ∼84 text messages	SNAP-Ed implementing agency delivered the intervention via text messaging. Participants recruited from local schools.	From pre- to posttest, parents reported increases in FV availability in the home, children’s FV consumption, and parents’ modeling FV consumption in the home.
Three lessons adapted from a SNAP-Ed Curriculum (e-health)	Neuenschwander [59], 2013	Measure the acceptability and test the effectiveness of online education compared with traditional in-person delivery to improve nutrition behavior.	SNAP-Ed eligible adult participants (*n* = 123)	4 wk; 3 nutrition lessons	SNAP-Ed paraprofessionals delivered online and in-person intervention. Participants recruited from SNAP-Ed site.	Most nutrition-related behavior outcomes improved from pre- to postintervention for both groups, but there was no difference in the magnitude of the change between groups.
VeggieBook [m-health (app)]	Clarke [27], 2019	Measure the acceptability and effectiveness of an app to promote the use of vegetables provided in meal preparation.	dyads of SNAP-eligible household cooks and one of their children (*n* = 286)	10 wk; user chose engagement	Research team delivered intervention at food pantries and via loaned smartphones. Participants recruited from food pantries.	App users were more likely to prepare “test vegetables” and to cook with a wider assortment of vegetables compared with nonusers.

Abbreviations: CM, Cooking Matters; FV, fruits and vegetables; SMS, short message service; SNAP, Supplemental Nutrition Assistance Program; SNAP-Ed, Supplemental Nutrition Assistance Program – Education; WIC, Special Supplemental Nutrition Program for Women, Infants, and Children.

**TABLE 5:** Summary of studies on e-health and m-health dietary interventions among SNAP and WIC participants

Intervention name (type)	Citation	Study objective	Sample characteristics	Dosage^1^	Intervention setting and delivery	Relevant findings
About Eating (e-health)	Lohse [54], 2015	Test the effectiveness of an online program to improve healthy meal planning on a budget.	Women with low income (*n* = 288)	no timeframe; ≥1 of 6 web-based lessons	Research team delivered intervention online. Participants were recruited from SNAP participation lists and WIC offices.	Online participants run out of food before the end of the month less often, increased use of nutrition facts labels, greater use of budgeting tools, more confidence to make healthy, affordable food and meal planning.
Affordable Flavors	Tripicchio [39], 2023	Measure the acceptability and test the effectiveness of an online Nutrition Education Program in improving mealtime practices in families using SNAP and WIC.	Parents receiving SNAP and/or WIC (*n* = 257)	8 wk; user chose engagement	The intervention was delivered online. Facebook and Instagram were used to promote engagement with the program. Participants were recruited nationally via social media.	High acceptability. Participants reported improvements in meal preparation practices, the diet quality of meals for children, and self-efficacy, and decreases in grocery spending, mealtime stress, and food.

Abbreviations: SNAP, Supplemental Nutrition Assistance Program; WIC, Special Supplemental Nutrition Program for Women, Infants, and Children.

Participants were recruited for the e-health interventions from WIC clinics (*n* = 10) or administrative lists (*n* = 1), mobile apps (*n* = 2), SNAP-Ed sites (*n* = 2), SNAP office (*n* = 1), outpatient clinics (*n* = 1), parenting support groups (*n* = 1), local Title I schools (*n* = 1), and the remaining from sites offering social services, such as Head Start programs, public libraries, safety-net clinics, Area Agencies on Aging, senior affordable housing, and food pantries (TABLE 3, TABLE 4, TABLE 5).

In the studies reviewed, there was a wide variety of modes of delivery and intervention settings (TABLE 3, TABLE 4, TABLE 5). For 4 WIC interventions, the e-health arm of the study was often developed and administered by research teams online, whereas control groups received usual care at WIC clinics by WIC staff [50,51,61,62], and for 2 interventions, researchers delivered both the in-clinic and online interventions [28,33,48,49]. Two WIC interventions without a control group were delivered via social media by researchers [40,50]. One WIC intervention was delivered by breastfeeding peer counselors via text messaging and in clinic for the control group [35,36,45], and 3 WIC text messaging interventions were delivered through third-party web-based platforms managed by research team members [34,52,53,60]. Two feasibility studies of WIC mobile apps were explored in a one-time in-person session (1 at the WIC clinic and 1 at participants’ homes) [41,47], and 2 of the evaluated e-health interventions were used by WIC state agencies as part of their standard practice (wichealth.org, WIC shopper app) [[29], [30], [31], [32],^37^].

In interventions intended for SNAP-eligible individuals and SNAP participants, researchers played a role in delivering online interventions through social media or websites [39,42,46,54], via text messages [38], and 2 interventions, whereas primarily conducted online by researchers, featured an in-person component for participants to collect produce at a pantry [27,43,44]. However, half of the interventions were designed for SNAP participants and were facilitated by public health practitioners. Extension and public health department staff facilitated 1 intervention through videoconferences and teleconferences [56]. For another intervention, SNAP-Ed and Expanded Food and Nutrition Education Program (EFNEP) staff were responsible for both in-person and remote delivery [57], whereas the SNAP-Ed implementing agency administered an intervention via text messaging [58]. Furthermore, SNAP-Ed paraprofessionals implemented 1 intervention online and in-person [59], and 1 nonprofit organization developed and administered the intervention via an app [55].

The methods of interventions also varied. Some interventions included online synchronous lessons using a streaming platform [56] or provided self-paced modules most similar in traditional nutrition education delivery [57,59,61,62]. Other interventions included more active involvement, such as in the case of 2-way texting [35,60] or a combination of texting, telehealth visits with healthcare providers, and community building via social media groups [50]. App studies allowed participants to engage with the app content freely. In one instance, the content of an intervention (Cooking Matters, CM) was delivered in 2 different ways: one study looked at the acceptability of the CM app [55] and one examined the effects in attitudes and behaviors for new followers of the CM Facebook page [40].

TABLE 3, TABLE 4, TABLE 5 show that dosage (e.g., length of intervention and amount of contact for each touchpoint) varied broadly across studies. App interventions allowed participants to choose the level of engagement in exploring the content to determine acceptability and effectiveness based on use of the app features. The median length for the interventions was 12 wk, with ≥4 interventions using engagement data offering no timeframe. These interventions included participants if they had completed ≥1 or 2 modules of the intervention or if they had downloaded the app (no level of use required). Texting interventions varied, with some sending 1 to 3 weekly messages and others sending hundreds of texts over 3 mo. Among the m-health interventions, 2 allowed for 2-way texting between peer counselors and participants [35,60].

### Effectiveness: dietary behaviors addressed in e-health intervention studies

Among studies evaluating the effectiveness of interventions aimed at WIC participants, 11 studies yielded positive outcomes [[29], [30], [31], [32], [33],^33^,[35], [36], [37],^49^,^50^,^61^], 3 yielded null results on all outcomes of interest [34,53,62], and 3 showed mixed results (e.g., some positive and some null results) [28,40,51]. As for studies intended for SNAP participants, 4 studies demonstrated positive effects [27,38,55,58], with 3 showing null results [44,57,59]. Both studies in SNAP and WIC participants had positive results [39,54]. The subsequent section presents the effectiveness of these interventions categorized by the dietary behaviors they addressed.

#### Food choice

Thirteen studies tested the effectiveness of the interventions on improving desired food choice outcomes with different approaches: increased WIC benefit redemption, promoted nutrient-dense food preparation, and increased skills in food resource management.

##### Choosing prescribed foods

An app to help participants redeem their prescribed WIC food packages (e.g., WICShopper app) showed high effectiveness in helping families choose more healthful DGA-based food benefits [31,32]. The WICShopper app facilitated household food choice by helping parents determine which products are WIC-approved and how many benefits remain in their account. App users were significantly more likely to redeem the prescribed foods than nonusers in most healthy food categories (except exempt infant formula, which is one of the most popular food benefits in the prescribed WIC food package) [31,32].

##### Nutrient-dense food preparation

The WIC Fresh Start intervention included 3 online modules with complementary farmers’ market vouchers for redemption. This study showed no differences in fruits and vegetables (FV) intake but did improve farmers’ market knowledge, attitudes and behaviors, and voucher redemption [28]. Comparably, Food eTalk and Food eTalk: Better U interventions provided 10 eLearning modules and 3 mo of fresh FV for SNAP-eligible individuals [[42], [43], [44]]. Although there were no differences in anthropometric outcomes or dietary quality pre- and postintervention, participants organically developed a virtual support network via text message by sharing encouragement, grocery shopping tips, and pictures of their meal preparation [43].

Two interventions promoting nutrient-dense food preparation found greater dietary quality. The VeggieBook intervention provided ‘test vegetables’ and a smartphone app with vegetable-based recipes, food tips, and no-cost strategies for making mealtimes healthier among SNAP-eligible adults [27]. This intervention found greater likelihood of using more FV in food preparation for app users compared with the control group. The Healthy Roots intervention used texting to promote the consumption of WIC-approved foods among participants and found greater consumption of leafy greens from pre- to postintervention [60].

##### Food resource management

Household food resource management interventions, the CM app and CM Facebook page, were tested on SNAP and WIC participants [40,55]. The CM app offered participants recipes and tools for creating shopping lists and meal planning. Individuals who downloaded the CM app and answered a survey reported positive attitudes and self-efficacy toward meal planning, but limited inferences can be made due to a self-selected sample: early adopters were more likely to be motivated to engage in these behaviors [55]. Nevertheless, the CM Facebook page provided content derived from the CM program, and new followers reported improved self-efficacy to cook healthy food with a limited budget after following the page for 2 mo [40].

For the About Eating intervention, participants reported running out of food before the end of the month less often (compared with both baseline and control groups), and participants reported increased use of nutrition facts labels to make food choices, use of a written spending plan for food, self-efficacy to manage money to make healthy food available, and frequency of meal planning to include all food groups [54]. The Affordable Flavors intervention, which included a 30-d meal plan with an online guide on how to prepare foods on a budget, found improvements in preparation meal practices, healthfulness of the meals served to children, and decreases in grocery spending, mealtime stress, and food insecurity [39]. Finally, an intervention that compared the effectiveness of 6 SNAP-Ed nutrition lessons delivered through face-to-face instruction and a distance education format revealed similar improvements in food resource management, food safety practices, and nutrient intake for both the distance education group and the conventional face-to-face group [57].

#### Dietary intake

Some interventions were aimed to improve dietary behaviors among different target audiences: 1 for adults, 2 for postpartum females to improve child feeding practices, and 1 for parents of school aged children. Three of these interventions were m-health and one e-health. These studies examined the effectiveness of dietary intake interventions and found differences by dosage, level of engagement, and study adherence.

##### Dosage and dietary intake intervention effectiveness

Two interventions tested among WIC participants aimed to improve dietary intake of infants and children by educating parents on breastfeeding and complementary feeding practices for infants and children [35,45]. These interventions provided different dosage approaches: the SMS intervention by UH and UPR texted females once a week for 18 wk (total of 18 SMS messages), whereas the Lactation Advice Through Texting Can Help (LATCH) intervention offered 2-way texting with a minimum of ∼170 1-way texts in 13 wk. The first intervention did not show differences between the SMS and the control group In breastfeeding, even if participants reported liking receiving the messages and their content. The LATCH intervention only showed differences in exclusive breastfeeding behavior among individuals with high level of engagement in 2-way texting with their breastfeeding peer counselors. These high engagement participants had the opportunity to discuss breastfeeding concerns and complications and get support in real time via SMS [45].

The Text2BHealthy intervention also had a high dosage, sending 2 to 3 weekly messages during the school year to SNAP-eligible parents of elementary aged children. From pre- to posttest, parents reported increases in FV availability in the home, children’s FV consumption, and parents’ modeling FV consumption in the home [58].

One intervention that adapted 3 modules from a SNAP-Ed curriculum, showed most nutrition-related behavior outcomes (e.g., fruit, vegetable, whole-grain intake, Nutrition Facts label use, breakfast, and meal planning frequency) improved from pre- to postintervention for both the experimental and the control group, and there was no difference in the magnitude of the change between the web-based and the in-person interventions [59].

#### Eating behaviors

Five eating behavior interventions addressed eating habits, portions, eating occasions, and dieting: 1 to promote breakfast intake among parents and children, 1 to address child eating practices, and 3 for weight management among females with overweight and obesity in different life stages (e.g., pregnant, postpartum, and adults). The modes of delivery and dosage widely ranged in these interventions, which may have impacted effectiveness.

Two interventions with high dosage and level of engagement from participants showed the most improvement for the intervention group. The Healthy4Baby intervention, which demonstrated greater weight loss among postpartum females in the intervention group, included 14 wk of daily text messages with personalized feedback communicating empirically supported behavior change strategies, daily skills, and self-monitoring, biweekly counseling calls from a health coach, incentives (e.g., raffles) to encourage self-monitoring, and access to a Facebook support group [50]. The MyQuest intervention for adult weight loss was successful in reducing body weight and other eating behavior outcomes and consisted of 168 to 252 texts and 12 electronic newsletters in 12 wk [38]. Low-dosage interventions, such as a single class to improve breakfast intake for parent and child, showed increases in breakfast frequency in the online group [61]. The SMS intervention for pregnant females sent a weekly message over 18 wk to promote healthy weight gain during pregnancy, and no differences were found between intervention and control groups [34].

The wichealth.org, a website used for 20 y by WIC to teach parents about best child feeding practices, included 8 modules to improve child eating practices [29,30,37]. The Internet group experienced substantial positive differences in stage of change progression, and FV consumption [37]. Traditional nutrition education required follow-up counseling to achieve FV consumption levels similar to the Internet nutrition education group [37]. A more recent study found that engagement with the wichealth.org materials varied by the type of device from which it was accessed (which may impact effectiveness of the intervention), with nonmobile users having greater engagement with the content compared with mobile users [30].

#### Multiple behaviors

##### Food choice and dietary intake for weight management

Two food choice and dietary intake interventions targeted weight management in the postpartum period: Fit Moms/Mamás Activas [[48], [49]] and the Smartloss app [51]. Both RCTs showed positive results for the intervention group, although the Smartloss effectiveness varied by level of treatment adherence. Both interventions had self-monitoring (e.g., weight log, pedometer) and a social support component (e.g., group meetings, health coach personalized calls).

### Acceptability of e-health dietary interventions

Studies found variable acceptability for e-health dietary interventions among SNAP and WIC adult participants. Some metrics for acceptability were study retention (SMS UPR), engagement with content as measured by key performance indicators (e.g., frequency and duration of sessions, frequency of using app features), and user feedback on perceived usability of the interventions through surveys and interviews [27,35,47,49,50,53,55,56,62].

Smartphone app users can engage with elements of the intervention at leisure, and may demonstrate or report a preference for certain features of the app. For example, users of the CM app reported that the recipe catalog feature was most often utilized for inspiration on how to cook in new and healthier ways, but the meal planning and grocery list were used less because users had created habits around using other methods of organizing these activities (e.g., writing down a shopping list). Similarly, Black and Hispanic WIC caregivers the who tested the Children Eating Well (CHEW app) reported high usability and perceived benefits for features that assisted them in making healthy food choices for their children (e.g., “Yummy Snack Gallery”) but reported lower ease of use and helpfulness for the barcode scanner, one of the WIC shopping tools to help redeem their prescribed WIC food package [47].

In another study, participants had split preferences for the method in which the materials were presented. In the VeggieBook app, a SNAP-Ed Toolkit intervention to increase familiarity with vegetable preparation, users compiled their favorite recipes through the app, and these were provided in paper format when participants visited the food pantry [27]. When asked about their preferences using VeggieBook materials in the kitchen, one-third of participants preferred using VeggieBook in the app, one-third preferred to use the printed version, and the rest had no strong preference for the format. However, 62% of the SNAP-eligible sample preferred to use their phones to view VeggieBook materials with their children.

Text/SMS interventions showed high acceptability as a method to deliver dietary information to postpartum females. Healthy Root m-health intervention participants reported that the texts with nutritional feedback (94%) and tips (87%) were helpful and would recommend the program (91%). The study evaluating an SMS intervention developed by UPR and UH to promote child feeding practices in postpartum females found acceptability was very good, as indicated by high participant retention (78%), a high rate of liking messages, reports of finding all messages useful in feeding infants, and success of messages in changing behaviors [52]. In the Healthy4Baby text intervention, most of the sample reported that the skills they learned in the program were extremely helpful (80%), found the text messages extremely useful (80%), and reported that the program was extremely successful in promoting weight control (100%). With the LATCH intervention, participants reported high satisfaction with the app (e.g., 91% would recommend app to peers) [35]. Survey data indicated that the messages were particularly helpful for first-time mothers or mothers who had not breastfed their prior children [35].

Three interventions tested the acceptability of adapted face-to-face curricula to digital delivery methods. The e-health intervention consisted of an online module that mimicked the delivery of the face-to-face education to reduce salt intake among WIC participants or caregivers, and there was high acceptability for the online intervention and similar outcomes for both delivery methods (e.g., reduction in salt intake of similar magnitude) [62]. Another study that tested acceptability was Fresh Conversations, which adapted 4 face-to-face lessons to live streaming-delivered interventions for SNAP-eligible older adults [56]. Overall, participants were “satisfied/very satisfied,” learned something new, and intended to make behavior changes, but there were no differences in group satisfaction or reported impacts for the groups that met through Zoom or Adobe Connect [56]. A household food choice study using a Facebook group to deliver an intervention adapted from a face-to-face curriculum, Food for Thought: Eating Well on a Budget, also showed high engagement and acceptability from a group of SNAP-eligible rural adults [46].

Changes in engagement with e-health/m-health dietary interventions are an indicator of intervention acceptability. The VeggieBook app users showed higher engagement from midstudy to the final measure, whereas the Fit Moms/Mamás Activas app intervention engagement decreased over time from 82% of participants logging in in the first month of the intervention to 17% in the last month [49]. In the Food eTalk study, participants organically initiated a text message thread to give each other support, take ownership of knowledge sharing, and problem solve [43]. Their engagement created new peer-to-peer elements to the intervention that were not originally incorporated in the design.

### Feasibility of e-health dietary interventions

The successful implementation of several interventions shows that e-health dietary interventions are possible to conduct among WIC or SNAP participants. Nevertheless, participants in various studies brought up technical issues as the main barriers to feasibility of e-health and m-health interventions. For example, with the CM app, phone data usage was a barrier to using the app in relevant places without Wi-Fi (e.g., grocery stores). For the CHEW app, many WIC users experienced technical barriers such as a broken phone, unsuccessful installation, and problems with features not working on their phone (e.g., the barcode scanner did not function well on certain Android phones). There were also issues with the accuracy of WIC-approved items database. The MomLink app delivered nutritional education to pregnant females [41], and although users reported some benefits (e.g., tracking pregnancy related information to show doctors, the information offered and the reminders to use resources), they also reported issues using the app. These issues included time constraints to use the app as intended, taking up space in their phones, unawareness of available features, and lack of interactivity with prenatal care providers (who had limited time to engage with participants within their workday) [41].

For the Fresh Conversations intervention, 25% of SNAP-eligible older adults reported technical difficulties logging into the monthly meeting, especially for the group with Adobe Connect [56]. Although several studies alluded to technical issues, only one study discussed motivation as the primary barrier to program feasibility [42]. Participants in the Food eTalk study recommended skill-based, visual education methods, such as cooking videos, recipes, and step-by-step teaching tools as a solution to increase motivation [42].

## Discussion

E-health interventions, which are comprised of varying modes including smartphone apps, web-based platforms, and text messaging services, have potential for helping individuals improve their food choice, eating behavior, dietary intake, and overall health outcomes (e.g., postpartum weight management). The current review aimed to investigate the effectiveness, acceptability, and feasibility of e-health interventions in improving dietary outcomes among SNAP participants, SNAP-eligible individuals, and WIC participants. The studies demonstrated e-health interventions’ effectiveness is associated with user engagement, study adherence and retention, and intervention dosage. Moreover, participants found digital dietary interventions to be acceptable, as they may reduce logistical barriers (e.g., coordinating childcare) and promote equitable access for individuals with limited resources (e.g., time constraints, reduced transportation access). In addition, some interventions could be relatively low-cost to implement (e.g., text/SMS interventions) showing feasibility and potential for scalability to federal nutrition assistance programs, such as SNAP and WIC. However, the review also identified challenges that impact the feasibility of implementing e-health interventions. Technical barriers, unmet expectations, and difficulties using technology were among the most cited challenges.

For the first objective of this paper, to determine effectiveness of e-health dietary interventions, the reviewers found that dosage, study adherence and retention, and engagement with content were important correlates or predictors of behavior change. Effective interventions with greater dosage, defined as the length of intervention, frequency, and amount of contact for each touchpoint, had multimodal touch points that included texting, personalized nutrition advice, and incentives to interact with content. However, it should be noted that providing personalized feedback may not be realistic for community nutrition educators in the federal nutrition assistance context. One of the studies reviewed that offered personalized care via an app found that WIC prenatal providers were not able to engage with participants as intended due to constrained time for study implementation [21].

The review findings indicate that the effectiveness of e-health interventions in improving dietary outcomes varied based on intervention dosage and frequency of participant exposure. Interventions with more frequent prompts [33,36,36,38,45,48,49,58] (e.g., >4 times per week), showed greater improvements in anthropometric measurements, self-efficacy to improve dietary behaviors, and FV intake compared with interventions with less frequent prompts [28,34,44,[51], [52], [53]], e.g., only once a week), even if the studies were comparable in length (13–18 wk). A previous review found that frequently prompting participants via notifications or text to complete tasks like self-monitoring and posting increases engagement, study retention, and efficacy [64]. Similarly, this review found that study adherence (i.e., extent to which participants followed the study recommendations), retention (i.e., percentage of participants who remained in the intervention until its completion), and engagement (i.e., level of involvement and interest that participants had in the intervention) were important predictors of effectiveness. To report retention, adherence, and engagement, studies used metadata from the apps such as link clicks, login attempts, number of times the app was opened, time spent using app, participation in study activities, and sustained text/SMS replies over the study period. For example, the Smartloss intervention found no overall differences in weight loss between intervention and control groups who received standard care; nevertheless, the group of mothers with high adherence to the intervention showed improved outcomes compared with the control group. Face-to-face dietary interventions with higher levels of retention have also shown long-term (e.g., >5 y) sustained improvements in dietary behaviors and health outcomes [65]. However, these types of studies often face challenges with retention and adherence, as participants may find it difficult to maintain the prescribed dietary recommendations for an extended period and so may “give up” if they feel they have not adhered well to a particular “diet.”

There may be additional health equity implications around study engagement, adherence, and retention. For instance, higher levels of engagement, adherence, and retention are associated with greater effectiveness of m-health dietary interventions, but inequalities in engagement by demographic characteristics may lead to high dropout rates for specific at-risk groups [66]. Ultimately, this may have a negative unintended consequence (e.g., those who most need the program may not be able to fully use it), and it also makes it difficult to draw conclusions about the effectiveness as well as any long-term sustainability of these interventions. The studies reviewed here did not discuss the effects of group-specific attrition. To improve engagement and study adherence, e-health interventions should evaluate disparities in engagement by demographic characteristics to tailor recruitment and retention approaches, maintain communication with participants via text messaging, push app notifications, and phone calls/emails throughout the study, and mimic typical smartphone and Internet browsing habits of individuals with low income [42]. Issues of fidelity in delivery were not discussed in the studies.

The second objective, examining the acceptability of e-health dietary interventions, found overall high acceptability among WIC and SNAP participants in the studies. A previous study among WIC participants examined Internet use preferences and found that participants liked to use the following: *1*) text messaging and online options for nutrition education; *2*) smartphone applications to identify WIC-approved foods; and *3*) a stronger Facebook presence for interacting with WIC clients and supporting breastfeeding [11]. Similarly, a study among vulnerable families found that digital media platforms had high acceptability when used to modify health behaviors [67]. Most families scored digital platforms highly in all acceptability domains [67]. However, acceptability likely varies by demographic characteristics, such as age, level of education, income, and racial/ethnic background [11]. A 2018 article on rural SNAP-Ed participants showed that slightly less than half young adults, half of middle-aged adults, and most older adults were not interested in online nutrition education [9]. Research has also found that cultural acceptability of the digital interventions among adults with food insecurity is critical for their successful implementation, which has strong health equity implications (e.g., a culturally unacceptable e-health intervention may get less engagement from participants who belong to racial and ethnic groups with higher nutrition-related chronic diseases) [68].

The COVID-19 pandemic highlighted the food and nutrition security disparities that Black, Indigenous, and people of color experience and the need for robust and equitable food assistance programming [69]. For this reason, in conjunction with modernizing the programs, the federal government is prioritizing equity in SNAP and WIC services [69]. E-health interventions that promote equity should be acceptable to all SNAP and WIC participants, which requires careful consideration of the cultural diversity and language needs of participants. Moreover, to promote equity, e-health interventions should be available to all SNAP and WIC participants. Yet, the state-based approach within WIC and SNAP-Ed and limited SNAP-Ed approach for SNAP participants may limit the reach and representativeness within these programs. The demographic diversity in the studies reviewed, although often reported, at times was not contextualized (e.g., lack of discussion about the implications of unbalanced attrition by race/ethnicity). In 2020, 13% of WIC and 20% SNAP participants were non-Hispanic Asian, non-Hispanic American-Indian or non-Hispanic multiracial participants, but no studies had participants from these populations [70,71]. Researchers’ failure to recruit a sample representative of the intended population or exclusion based on convenience (e.g., English-language mastery as an exclusion criterion) can have negative unintended impacts on studies’ generalizability, reproducibility, and acceptability for groups who did not test the intervention. Further, few studies discussed cultural tailoring of interventions. Some strategies to make an intervention more acceptable included translation of language or modification of recipes for Spanish-speaking populations or non-White participants. Tailoring also needs to be both applied and described in the research methods to ensure more replicable and generalizable public health programming.

Another important factor that can hinder health equity in e-health intervention implementation is a lack of consideration for the health and digital literacy of the intended audiences. A scoping review on interventions to guide healthy choices in the online food retail environment noted that health and digital literacy is seldom considered and never measured in e-health studies [72]. The present review also found that none of the studies examined measured the health literacy of the participants. To enhance health equity, implementers of e-health dietary interventions for SNAP and WIC participants should address the digital health literacy of the participants [73]. New studies should be more explicit about culturally tailoring the interventions and matching educational content to the digital health literacy of participants to improve the acceptability (e.g., usefulness, convenience, understandability, and enjoyability) of the interventions.

The third objective, feasibility, explored how possible e-health interventions were to implement with WIC and SNAP audiences. All the studies examining feasibility found that they were able to reach most participants using e-health channels [12,17,21,33,35,51]. Moreover, throughout the pandemic, federal nutrition programs embraced text messaging and other online communications to maintain services; therefore, the infrastructure to deliver e-health interventions has been strengthened in the past few years. Still, technical problems and Internet access were most often discussed in studies as the main barriers to feasibility. There are disparities in access to devices, with 57% of lower income individuals having a broadband connection at home and 27% relying solely on smartphones for Internet access [12,13]. The implications of low technology access can impact long-term use of any type of intervention. Even with the documented effectiveness of the wichealth.org website in the early 2000s, a study in 2016 found that there were differences in participant engagement with the lessons depending on the type of device utilized to access the intervention. Considering the disparities in broadband connection and the reliance on smartphones for Internet access for over one-quarter of individuals with annual incomes under $30,000 [12,13], some inequities in engagement can potentially impact the feasibility of the tested e-health dietary intervention for individuals with lower income.

For subsets of the population, Internet disparities could further health inequity issues. For example, Black and Hispanic households are less likely to own a computer or have Internet broadband, rural Americans are less likely to have access to a smartphone and home broadband access compared with Americans in urban and suburban areas, and although older Americans have more access to Internet than 10 y ago, significant disparities remain [[74], [75], [76]]. A study in Alaskan Native WIC participants found that barriers to potentially engaging with nutrition education online included slow Internet, no computer access, and the high cost of Internet service [49]. Nonetheless, e-health interventions can reduce time and transportation barriers for federal food assistance participants and effectively support health equity for these populations.

The present review exhibits both strengths and limitations. As a strength, this review offers a comprehensive examination of existing research, allowing for the synthesis of evidence from multiple studies by dietary behaviors. This synthesis will allow researchers and public health practitioners to make well-informed decisions and recommendations regarding the implementation of e-health dietary interventions tailored to the prioritized dietary behaviors of the populations they serve. However, several limitations need acknowledgment. First, the incorporation of diverse outcome metrics across studies posed challenges in making meaningful comparisons and consolidating results. Furthermore, the quality of some RCTs were lower due to the inability to blind participants and assessors to the treatment type, the absence of attrition analysis based on demographic characteristics, and a lack of discussion concerning the randomization structure. Additionally, in certain instances, interventions involving SNAP participants or SNAP-eligible individuals were not administered by SNAP-Ed staff [27,38,42,46,55], leaving uncertainty about their feasibility within SNAP-Ed implementing agencies. Finally, there is a risk for publication bias, favoring the publication of positive results, which can potentially influence the overall findings of the review. Nevertheless, e-health interventions demand substantial resources; therefore, reporting neutral and negative outcomes is deemed likely.

### Implications for research and practice

E-health interventions, including smartphone apps, web-based platforms, and text messaging services, can help individuals improve their dietary habits and overall health. For SNAP participants, SNAP-eligible individuals, and WIC participants receiving virtual food and nutrition education, it can be effective, acceptable, and feasible. E-health advances contribute to making SNAP-Ed and WIC online food and nutrition education more interactive, innovative, and fun. Some of the most effective studies reviewed had frequent and multimodal contacts with participants throughout the intervention period. However, the real-time personalized nutrition feedback some interventions explored could increase burden for program staff, making it unsustainable. Overburdening public health nutrition providers limits feasibility overall and could reduce the adoption, implementation, and maintenance of e-health dietary interventions. For interventions that involve healthcare providers or nutrition educators, practitioners should ensure that these professionals have the necessary time and resources to engage effectively with participants. Finding a balance between the level of personalization and implementation burden for staff can help optimize the reach and impact of the interventions.

In addition, considerations about digital literacy and access, cultural tailoring, and language accommodations should be prioritized when developing e-health interventions for federal nutrition assistance participants. To align with the federal government objectives on modernization and health equity, practitioners and researchers involved in implementing e-health interventions among food assistance participants should ensure that interventions are culturally appropriate, accessible, and acceptable to all participants. Future studies should also consider the disparities in technology access and design interventions that can be effectively delivered to individuals with lower incomes and limited access to devices and Internet connectivity.

Federal and state agencies have embraced the use of digital tools to deliver food and nutrition education and services. For example, a review found 17 available apps for use in the WIC program with wichealth.org and the WIC shopper app were available in most states [77]. Moreover, e-health dietary interventions for food assistance participants are proliferating, with several protocols for RCTs aimed to improve health outcomes for postpartum females in WIC and children in SNAP-eligible families (e.g., HomeStyles2, SmartMoms app) [78,79]. To maximize their impact, future research and practice should focus on tailoring interventions to the diverse needs of the target population, addressing disparities in engagement and access to technology, and ensuring cultural appropriateness and health equity in intervention design and implementation. Additionally, assessing other implementation outcomes, such as long-term sustainability, cost effectiveness, and provider acceptability, will contribute to the successful integration of e-health interventions into federal nutrition assistance programs.

### Author contributions

The authors’ responsibilities were as follows – CBS, AY: conceived research; MCB, CBS: designed research; MCB, CBS: conducted research, extracted data, and prepared the first draft of the manuscript; MCB, JFO: conducted the selection and evaluation of studies; and all authors: revised, read, and approved the final manuscript.

### Conflict of interest

The authors report no conflicts of interest.

### Funding

Research reported in this publication was supported by the National Institute of Diabetes and Digestive and Kidney Diseases (NIDDK), the Office of Disease Prevention (ODP), the Office of Nutrition Research (ONR), the Chief Officer for Scientific Workforce Diversity (COSWD), and the Office of Behavioral and Social Sciences Research (OBSSR) of the National Institutes of Health under award number (NIDDK U24 DK132733). The content is solely the responsibility of the authors and does not necessarily represent the official views of the National Institutes of Health.
